# The Effect of Welding Defects on the Long-Term Performance of HDPE Pipes

**DOI:** 10.3390/polym14193936

**Published:** 2022-09-21

**Authors:** Huansheng Lai, Dengshuai Fan, Kanglin Liu

**Affiliations:** 1 Sino-French Institute of Nuclear Engineering and Technology, Sun Yat-sen University, Zhuhai 519082, China; 2 School of Mechanical and Power Engineering, Nanjing Tech University, Nanjing 211816, China; 3 School of Chemical Engineering, Fuzhou University, Fuzhou 350116, China

**Keywords:** HDPE, creep life, welding defect, butt fusion joint

## Abstract

High-density polyethylene (HDPE) pipes are the preferred pipes of water systems in nuclear power plants because they are durable, corrosion-free, easy to install, and not subject to fouling. However, their long-term performance can be affected by welding defects. In this paper, the effect of welding defects on the long-term performance of HDPE pipe butt fusion joints was studied using a creep test. A welding defect with a hole or inclusion in the joint was simulated by artificially inserting a copper ball during butt fusion welding. The test results showed that the creep life of the joint decreased with increased defect size. An expression describing the creep life and the defect ratio was obtained according to the test results. In addition, the test results showed that the creep life of the joint without a welding bead was about 50% of that in a joint with a welding bead.

## 1. Introduction

High-density polyethylene (HDPE) pipes are widely used in oil and water systems due to their ease of installation, long service life, good corrosion resistance, environmental protection ability, and excellent mechanical properties [1]. HDPE pipes have also been proven to be better than metal pipes for use in water systems in nuclear power plants [2]. In order to ensure the water-tightness and integrity of a piping system, HDPE pipes are usually required to be welded. The main welding methods for HDPE pipes are butt fusion welding and electrofusion welding. In the case of large-diameter HDPE piping systems in nuclear power plants, butt fusion welding is the most effective and most commonly used method [3]. For butt fusion welding, the two end surfaces of the welded pipe should first be milled with a milling cutter. After that, the two surfaces are heated using a heating plate and they melt under pressure. Then, the two surfaces are quickly connected together under pressure. Finally, the butt fusion joint is cooled to an ambient temperature under pressure. The performance of butt fusion joints is mainly affected by certain welding parameters, such as temperature [4,5], pressure [6,7], and heating time [8]. In addition, axial misalignment of the joint can reduce the service life of butt fusion joints [9]. Furthermore, the performance of butt fusion joints is also affected by welding defects, such as porosity, inclusions, and incomplete fusion. Therefore, butt fusion joints are the weakest link in an HDPE piping system.

With regard to the short-term performance of polyethylene (PE) pipes, studies have shown that there is no significant difference in the tensile properties between the base material and the butt fusion joint [10,11]. Furthermore, it was also found that the yield strength [12], burst strength [13], bending performance, and crush performance [14] were almost unaffected by a welding defect when the defect size was smaller than 15% of the wall thickness of the pipe.

Studies have also been carried out on the long-term performance of PE pipes, such as creep life, long-term hydrostatic strength, and slow crack growth resistance [15,16,17,18]. The authors have found that the slow crack growth resistance of butt fusion joints is weaker than that of the base materials and that the allowable size of welding defects should be investigated [19]. A study indicated that the stress concentration at the junction between the welding bead and the pipe’s surface is not harmful to the creep life of pipes in the case of ductile failure [3]. Another study showed that the effect of Vaseline and graphite contaminants on the performance of butt fusion joints would be negligible at high welding pressures, but Teflon contamination on the welding surface can significantly reduce the service life; this phenomenon was more significant at low welding pressures [20]. In addition, test results have indicated that the creep test was more effective than the hydrostatic strength test in evaluating the effect of welding defects on the long-term performance of PE pipes [21]. However, up to now, the effect of welding defects, such as holes or inclusions, on the long-term performance of butt fusion joints has not been clear, even though limited studies have been carried out in this field [21,22].

In this study, the effect of welding defects on the long-term performance of HDPE pipe butt fusion joints was studied using a creep test. Copper balls were inserted into the joints of HDPE pipes to simulate the welding defects of holes or inclusions. Based on the test results, the effect of defect size on creep life was discussed and a relationship between the creep life and the defect size was obtained.

## 2. Experimental Procedure

**Figure 1:**
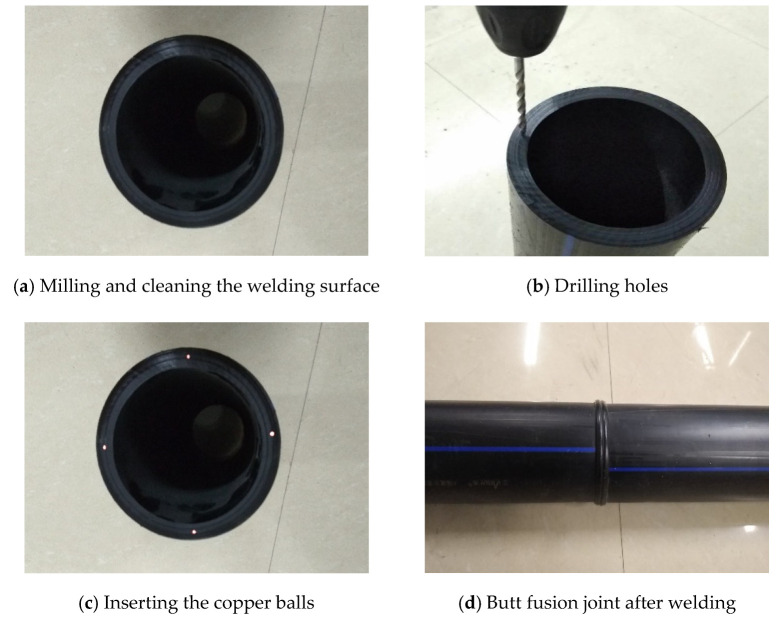
Process of inserting defects into the butt fusion joint of HDPE pipes.

HDPE pipes made from PE100 were used in this study. They had an outer diameter of 200 mm and SDR (the ratio of the outer diameter to the wall thickness) of 11. The melting temperature, *T*_m_, was 131 °C, the crystallization temperature, *T*_c_, was 117 °C, and the degree of crystallinity was 67%. The yield stress was 22.2 MPa, the Poisson’s ratio was 0.4, and Young’s modulus was 1314 MPa at an air temperature of 28 °C. The HDPE pipes were welded according to ISO 21307 [23] and the welding parameters are shown in Table 1. The welding was performed on a butt fusion welding machine (HDC160–315, Zhuji Huida Pipeline Technology Co. Ltd., Zhuji, China). More details on the welding process can be found in ISO 21307 [23]. Copper balls were inserted into the welding surface before welding in order to simulate welding defects such as holes or inclusions. The steps of inserting the copper balls are shown in Figure 1a: milling and cleaning the welding surface; Figure 1b drilling holes in the center of the welding surface (the diameter of the holes was identical to that of the copper balls); Figure 1c inserting the copper balls; Figure 1d creating the butt fusion joint after welding. The ratios of the diameter of the copper balls to the thickness of the pipe wall were 0%, 16%, 33%, and 44%, respectively. Here, 0% indicates that there was no welding defect in the joint. Four copper balls were inserted into the joint to prepare enough specimens in one joint and reduce the effect of welding quality on the test results. The defect sizes are listed in Table 2.

**Figure 2:**
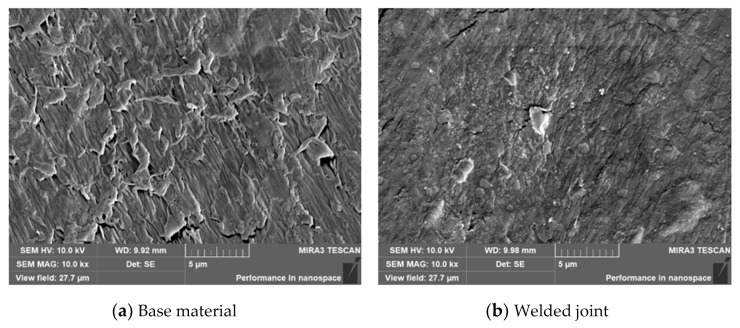
SEM micrographs of the base material and welded joint of HDPE.

Samples were prepared from the base material and the welded joint of the HDPE pipe to research their microstructure. The samples were polished using an ion beam and then analyzed using a scanning electron microscope (SEM, TESCAN MIRA 3, Brno, Czech Republic). The results are shown in Figure 2. As shown in Figure 2a, there are numerous lamellae in the base material. However, the lamellae were almost not present in the welded joint, as shown in Figure 2b. Therefore, it could be deduced that the weld joint was harder and more brittle than the base material.

**Figure 3:**
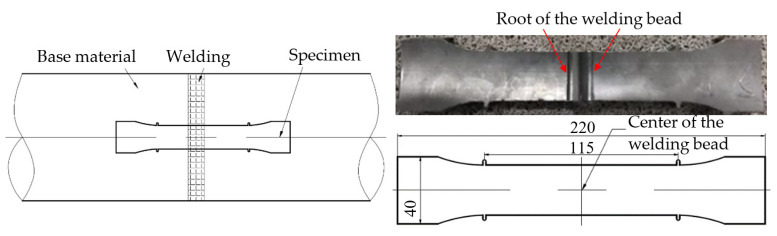
Processing method and dimensions of the creep specimen.

The creep specimens were processed according to EN 12814–3 [24]. The welding bead was located in the middle of the specimen and the welding defect was in the center of the welding bead. The processing method and dimensions of the creep specimens are shown in Figure 3.

All tests were performed on a creep-testing machine (DKRE50, Delitest Co. Ltd., Changchun, China) equipped with an environmental chamber. All tests were carried out in the room’s air and at a temperature of 40 °C. The temperature was controlled by the environmental chamber, with a temperature error of ±1 °C. A stress of 9 MPa was applied to all specimens and the load line displacement at the gauge length, as shown in Figure 3, was recorded during the tests. Note that the gauge length was 115 mm, as shown in Figure 3. Three specimens were tested for each defect size; hence, a total of 12 specimens were tested. In addition, for specimens with no welding defect, another two specimens, e.g., one with a welding bead and the other one without a welding bead, were tested at a temperature of 28 °C in order to research the effect of the welding bead on the creep life of the joint.

## 3. Results and Discussion

**Figure 4:**
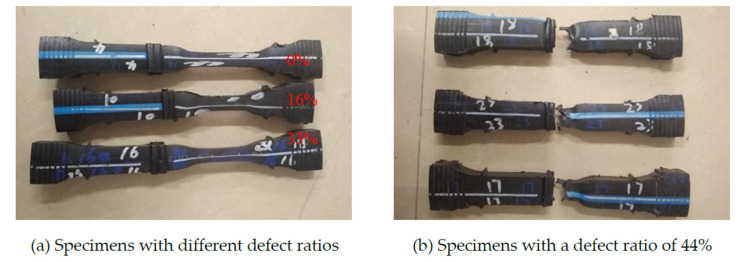
Creep failure specimens of HDPE under a load of 9 MPa and at a temperature of 40 °C.

For specimens with a welding bead, the experimental results showed that the specimens with defect ratios of 0%, 16% and 33% necked in the base material, as shown in Figure 4a. The reason for this was that the stress concentration in the root of the welding bead, as shown in Figure 3, did not damage the ductile failure of the joint [3] and the joint region was harder than the base material. Therefore, necking appeared in the base material. However, a brittle fracture was found to occur at the joint for all specimens with a defect ratio of 44%, as shown in Figure 4b. One reason was that the joint was subjected to greater stress than the base material because the defect ratio of 44% significantly reduced the cross-sectional area of the joint.

**Figure 5:**
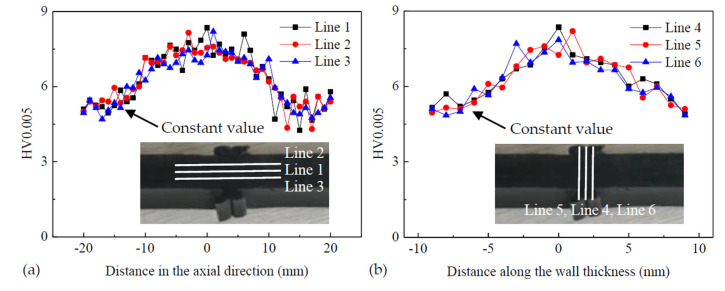
Distribution of hardness in the butt fusion joint of the HDPE: (**a**) hardness in the axial direction, (**b**) hardness along the wall thickness.

The Vickers hardness was tested to research the distribution of hardness in the HDPE pipe’s welded joint. The hardness was tested using a hardmeter (HV-1000Z, Shanghai Precision Instruments Co. Ltd., Shanghai, China). Tests were carried out at air room temperature (25 °C). A load of 0.005 kgf was applied and held for 10 s during the test. The values of the hardness of the joint are shown in Figure 5, where the distance of 0 mm means that the hardness was measured in the center of the joint or the pipe wall. The hardness was measured in the axial direction (lines 1–3: from left to right) and the thickness direction (lines 4–6: from the bottom up) respectively, as shown in Figure 5. Line 1 was located in the center of the thickness of the wall and line 5 was located in the center of the joint; the distance between the two lines was 1 mm. As shown in Figure 5a, the greatest hardness was located in the middle of the joint and then the hardness slowly decreased to a constant value in the axial direction. A constant value of hardness appeared in the base material, at about 20 mm away from the middle of the joint. The highest value of hardness was about 1.7 times larger than the constant value. The hardness slowly increased to the largest value and then decreased from the inside to the outside of the thickness of the wall, as shown in Figure 5b. The reason was that the material was squeezed into the two sides of the pipe wall under pressure during the welding process. In this way, the material in the middle of the wall was subjected to the greatest pressure. Therefore, the center of the joint had the highest value of hardness. This means that the center of the joint was more brittle than other regions and is the reason why a brittle fracture appeared in the joint, as shown in Figure 4b.

**Figure 6:**
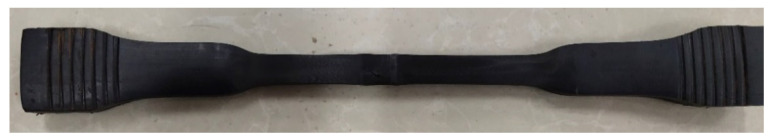
Creep failure specimen of HDPE without welding bead and welding defect under a load of 10 MPa and at a temperature of 28 °C.

When the welding bead was removed, the specimen necked in the welding seam and the base material, as shown in Figure 6. The reason was that there was no welding bead to strengthen the joint. This phenomenon was quite different from the specimen with the welding bead, as shown in Figure 4a. Therefore, the creep failure of the specimen was affected by the welding bead.

**Figure 7:**
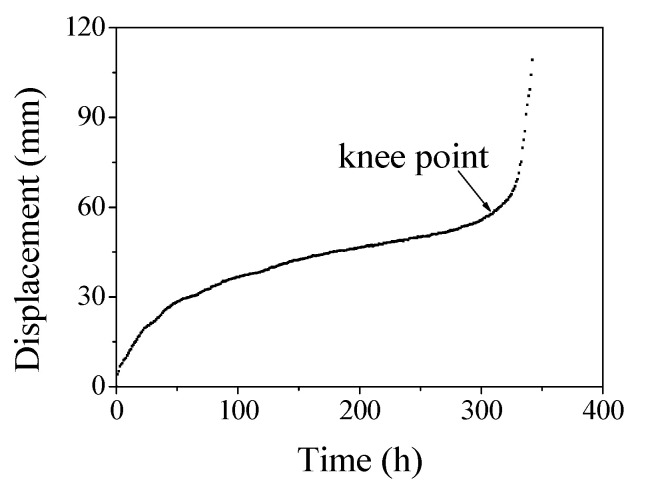
Variation of displacement with time for a specimen with a defect ratio of 33%.

Since an HDPE pipe has good plasticity, the specimens did not fracture during the tests when the defect ratios were less than 44%, even though the necking deformation was very significant, as shown in Figure 4a. Therefore, the creep life was defined at the knee point, where the displacement rapidly increased as the creep time increased in this study, as shown in Figure 7. The creep life of each specimen is shown in Table 3.

**Figure 8:**
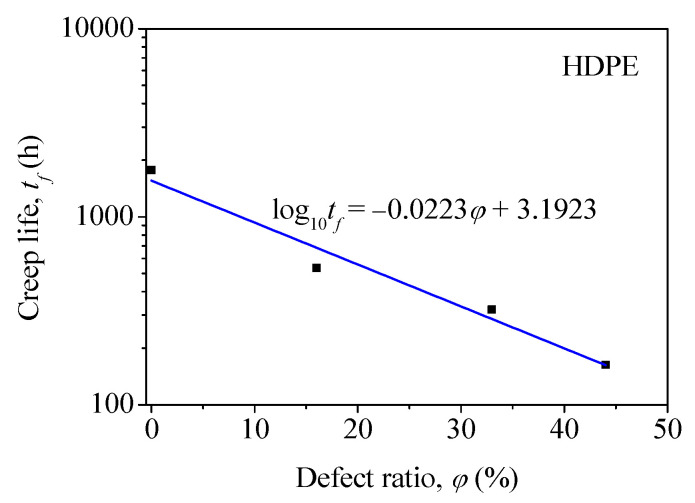
Variation of creep life with the defect ratio for the HDPE specimen under a load of 9 MPa and at a temperature of 40 °C.

The variation in creep life with the defect ratio is shown in Figure 8, where each value of creep life was the average value of three specimens. As shown in Figure 8, the creep life, $t_{f}$, decreased almost linearly with the increased defect ratio, *φ*. Therefore, a linear curve between the creep life and the defect ratio was fitted, which is represented by the blue line shown in Figure 8. The expression of the fitted curve was shown in Equation (1).

(1) $$\mathrm{lg}t_{f}=-0.0223\varphi +3.1923$$

For the specimen without a welding bead, the creep life was only about 50% of the specimen with the welding bead, as shown in Table 3, where the test was conducted at a temperature of 28 °C; the two specimens had no welding defect in the joint and underwent the same load of 10 MPa. The reason was that the specimen failed first in the joint and then the failure extended to the base materials, as shown in Figure 7. As we know from previous studies, the creep resistance of the joint was weaker than the base material [19]. As a result, the creep life decreased significantly after the welding bead was removed. Therefore, the welding bead was beneficial to the creep life of the joint in the case of ductile failure.

In addition, the obtained Equation (1) can be used to predict the creep life of the HDPE pipe butt fusion joints with a welding defect in the form of a hole or inclusion. However, it can only be used with a test temperature of 40 °C and an applied stress of 9 MPa. Therefore, further work needs to be carried out in order to study the effect of welding defects on the long-term performance of HDPE pipe butt fusion joints at different stresses and temperatures. Furthermore, the creep properties of the joint need to be studied further in order to establish the exact creep resistance of the joint. In this way, the long-term performance of the HDPE pipes used for water systems in nuclear power plants can be accurately predicted.

## 4. Conclusions

In this paper, the effect of welding defects on the long-term performance of HDPE pipe butt fusion joints was studied using a creep test. The main conclusions were as follows:

(1) The results showed that the highest hardness level in the butt fusion joint of HDPE pipes was 1.7 times greater than in the base material. The greatest hardness was found to be located at the center of the welded joint.
(2) The creep life of butt fusion joints of HDPE pipes was affected by the welding defect and welding bead. As the size of the welding defect increased, the creep life significantly decreased. The creep life of the specimen without a welding bead was about 50% of the specimen with a welding bead.
(3) An equation was obtained from the experiment results to describe the relationship between the defect ratio and the creep life of the HDPE pipe butt fusion joints. This can be used to predict the creep life of the HDPE pipe butt fusion joints with a welding defect such as a hole or inclusion.

## Figures and Tables

**Table 1:** Welding parameters of the HDPE pipe butt fusion joints [23].

Heating Temperature (°C)	Endothermic Time (s)	Cooling Time (Min)	Flanging Pressure (MPa)	Welding Pressure (MPa)
210	150	17	0.15	0.15

**Table 2:** The defect diameter, wall thickness, defect ratio and creep life of the specimens.

Case	Defect Diameter (mm)	Wall Thickness of the Pipe (mm)	Defect Ratio (%) (Diameter/Thickness)	Creep Life $t_{f}$ (h)	Average Creep Life (h)
1	0	18.2	0%	1803	1771
2				1732	
3				1779	
4	3	18.2	16%	529	534
5				511	
6				562	
7	6	18.2	33%	305	321
8				317	
9				342	
10	8	18.2	44%	152	163
11				178	
12				159	

**Table 3:** Creep life of HDPE specimens under a load of 10 MPa and at a test temperature of 28 °C (with no welding defect in the specimen).

Specimen	Creep Life $t_{f}$ (h)
With welding bead	2885
Without welding bead	1392

## Data Availability

Not applicable.
